# Examining the Relationship Between Pediatric Clerkship Timing and Performance: COVID-19 Pandemic and Beyond

**DOI:** 10.1007/s40670-025-02339-2

**Published:** 2025-03-04

**Authors:** Meaghan S. Wido, Alyssa L. MacMahon, Steven J. Durning, Ting Dong, Sami A. Abuhamdeh

**Affiliations:** 1 Department of Pediatrics, F. Edward Hébert School of Medicine, Uniformed Services University, Bethesda, MD 20814 USA; 2 Department of Health Professions Education, F. Edward Hébert School of Medicine, Uniformed Services University, Bethesda, MD 20814 USA

**Keywords:** Pediatric clerkships, Medical education, Student performance, COVID-19, Multi-site

## Abstract

**Introduction:**

Medical education experienced significant changes over the past 5 years, with the COVID-19 pandemic having a considerable impact on students’ clinical rotations. The Uniformed Services University (USU) pediatric clerkship, which sends medical students to 11 geographically diverse clerkships sites, was no exception. This study sought to determine if there is a relationship between the COVID-19 pandemic and medical students’ performance outcomes in their pediatric clerkship. Sub-analyses sought to determine if these relationships vary by the clerkship site or timing within the academic year.

**Methods:**

Data included mean National Board of Medical Examiners® (NBME) scores from pre-clerkship, and NBME Pediatrics Subject Examination and History and Physical Exam (H&P) scores from the USU medical students who completed the pediatric clerkship during 2018–2023 (*N* = 978). Statistical analyses examined the relationship between students’ performance and timing, as well as performance across clerkship sites.

**Results:**

Students’ NBME scores were significantly lower in 2020 compared to 2018 (*p* < 0.001), 2019 (*p* < 0.001), and 2023 (*p* < 0.05). H&P scores differed significantly by year, but increased rather than decreased during 2020. There was a positive linear relationship between timing during the academic year and performance outcomes, which was more significant in and consistent across sites for NBME scores.

**Conclusion:**

Changes made to the clerkship during the COVID-19 pandemic (2020) may have impacted students’ performance on the NBME, but did not appear to impact their H&P scores. Sub-analyses suggest the experiences students have throughout the clerkship year may improve their overall performance.

**Supplementary Information:**

The online version contains supplementary material available at 10.1007/s40670-025-02339-2.

## Introduction

As the COVID-19 pandemic unfolded, offices, schools, and universities around the world closed. Quarantine guidelines in the USA decreased the number of in-person acute care visits [1] and necessitated an overall change in how medical students engaged in their clerkship experiences [2–5]. A survey of internal medicine clerkship directors from 137 Liaison Committee on Medical Education (LCME) accredited medical schools found that medical students were removed from direct patient care for a median of 85.5 days during the 2020 school year [6]. While removed from direct patient care, many medical students and preceptors transitioned to online learning, in which they relied heavily on simulated clinical experiences to supplement clerkships [7]. As medical students returned to their clerkship sites in late 2020 and early 2021, many experienced atypical clinical environments with increased virtual patient encounters, decreased face to face patient time, fewer preventive health visits, and shortened or modified clerkship rotations. In light of these shifts in clinical experiences during COVID-19, it is crucial to examine the resulting effects on medical students’ performance outcomes.

Emerging research has demonstrated mixed results when comparing medical students’ performance outcomes before and during the COVID-19 pandemic [8]. While some studies showed that there was a decrease in medical students’ performance during the pandemic [9], others demonstrated that there was no change in assessment scores [10–12] and some studies even showed an increase in exam scores [13]. With such a wide variety of results, each medical school may have its own environmental, systematic, and/or educational factors that contribute to differences, or lack thereof, in how students performed before, during, and after the pandemic. By looking at medical students’ performance outcomes during their pediatric clerkship, this study seeks to take the first step in understanding what these factors may be for the Uniformed Services University (USU).

One potential environmental factor unique to the USU is the geographic diversity of the clerkship sites. The USU is the nation’s only medical school dedicated specifically to the training of uniformed physicians in the Air Force, Army, Navy, U.S. Public Health Service, and U.S. Coast Guard. Consequently, and unlike most civilian medical schools, USU clerkship sites are located at geographically diverse Army, Navy, and Air Force military treatment facilities. In the pediatric clerkship, students are assigned to one of 11 sites spanning the continental United States and Hawaii. And while recent research has shown that the USU pediatric clerkship was able to maintain intersite consistency in students’ performance outcomes from 2013 to 2017 [14], this has not been investigated since the pandemic [15]. Additionally, as civilian medical schools are forced to adopt more multi-site clinical rotations, this study may serve as a guide for continued research in how medical schools adhere to the LCME Standard 8.7 Compatibility of Education/Assessment.

Prior to the COVID-19 pandemic, each clerkship rotation lasted 5 weeks. Similar to many civilian medical schools, the 12-month traditional block clerkship (referred to as “rounds” at the USU) divides students’ time between six core clerkships and one selective clerkship. For example, a student may spend 5 weeks with family medicine during “Round 1,” pediatrics during “Round 2,” psychiatry during “Round 3,” and so on. At the onset of the pandemic in March 2020, the USU clerkships were suspended. From March 2020 to June 2020, students participated in 2.5-day distance learning (i.e., virtual) modules for each of the specialties, with subject-specific didactics and clinical skills training. Clerkships resumed in-person on June 8, 2020, but were shortened to 4 weeks per round, removing the option for selective. In January 2021, clerkships returned to 5-week rotations, still with no option for a selective and additional time allotted for quarantining. Not until January 2022 did the clerkship return to its pre-pandemic schedule. In light of the many disruptions and changes to clerkships, this study examines relationships between USU medical students’ clinical experiences and performance in their pediatric clerkship. This study is guided by the following research questions:

1. Do USU medical students in their pediatric clerkship have similar clinical knowledge before, during, and after the COVID-19 pandemic as evidenced by National Board of Medical Examiners® (NBME) and written History and Physical (H&P) examinations?
2. What relationships exist between the time (i.e., round) students complete their pediatric clerkship and their performance outcomes?
3. How do these relationships vary by clerkship site?

We hypothesized that there would be a decrease in students’ performance on the NBME and written H&P at the height of the pandemic (i.e., 2020), and that performance would return to pre-pandemic levels no later than 2022. We additionally hypothesized that students’ performance would increase each round and this would not be impacted by timing of the pandemic. Lastly, we hypothesized that students’ performance would be similar at each clerkship site.

## Methods

### Context

The USU School of Medicine 4-year program consists of 18 months at the USU campus in Bethesda, MD, where students are enrolled in a pre-clerkship curriculum consisting of seven systems-based modules. Each systems-based module is concluded with a NBME basic science subject exam. Students spend the following calendar year completing their core clerkships at various sites, utilizing a traditional block clerkship model (referred to as “rounds” at the USU). Students attend the 5-week pediatric clerkship during one of nine rounds throughout the calendar year. During the clerkship, students must submit a H&P written report that requires them to complete an in-depth analysis of a pediatric patient from the inpatient ward. At the end of their pediatric clerkship, students take the NBME Pediatrics Subject Examination.

### Study Participants and Measures

The USU pediatric clerkship measures students’ performance based on a number of factors. For the purposes of this 5-year retrospective study, we are analyzing NBME Pediatrics Subject Examination scores and H&P scores of USU medical students who completed their pediatric clerkship between 2018 and 2023 (*N* = 978). These data were collected from the USU Department of Pediatrics archival students records. Mean pre-clerkship NBME score served as a control variable, and was the mean of each participant’s midterm and final exam scores of seven systems-based modules. In a small number of cases (*n* = 22), students had one or more exam scores missing, and were therefore not included in the sample. This study is a part of the Long-Term Career Outcome Study (LTOCS) approved by the USU Institutional Review Board (protocol number DBS.2020.076).

The NBME Pediatrics Subject Examination is a standardized, timed (2 h and 45 min), 110 multiple-choice assessment with a minimum passing score of 59. The H&P exam is assessed by site directors (i.e., the preceptors at each clerkship site in charge of grading students’ H&Ps) using the Pediatric History and Physical Exam Evaluation (P-HAPEE) 50-point analytic rubric (Appendix A) which has validity evidence.

### Statistical Analysis

An analysis of covariance (ANCOVA) was conducted to examine the relationship between year and NBME scores while controlling for pre-clerkship NBME scores. A second ANCOVA examined the relationship between year and H&P scores, controlling for pre-clerkship NBME scores. Follow-up analyses explored the relationship between performance across years and by site. The 6 years were grouped into three periods: Period 1 reflective of a regular clerkship schedule (2018 and 2019), period 2 reflective of an adapted clerkship schedule for COVID (2020 and 2021), and period 3 reflective of a return to regular clerkship schedule (2022 and 2023). Marginal means for each period at each of the eleven sites were estimated and compared with the overall marginal means. A regression analysis was conducted to examine the relationship between Round and NBME scores, controlling for pre-clerkship NBME scores. A second regression analysis examined the relationship between Round and H&P scores, controlling for pre-clerkship NBME scores. Follow-up analyses investigated the relationship between Rounds and performance (i.e., NBME and H&P scores) at each site. To achieve an adequate power level, the nine rounds were consolidated into three groups: Rounds 1–3, Rounds 4–6, and Rounds 7–9. That is to say, all students who completed their pediatric clerkship between 2018 and 2023 in Rounds 1, 2, or 3 were grouped together. Marginal means of performance outcomes for each of these three groups were estimated for all 11 sites and compared to the overall marginal means. A final set of exploratory analyses investigated potential interactive effects between Year and Round on performance. All statistical analyses were performed using R (version 4.3.3, R Foundation for Statistical Computing, Vienna, Austria).

## Results

### The Relationship Between Year and Performance

**Fig. 1:**
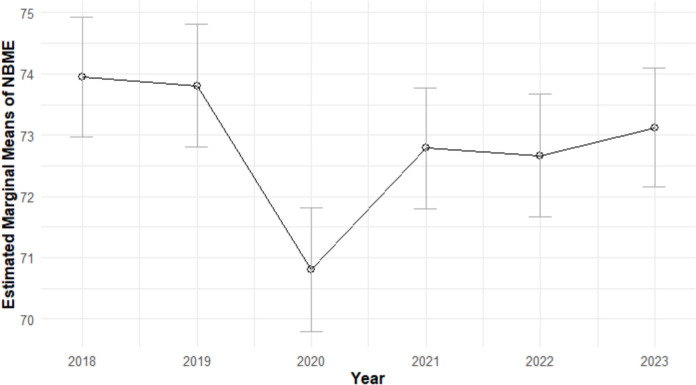
NBME means by year with 95% confidence intervals

Results of the ANCOVA examining the relationship between year and NBME indicated that NBME scores differed significantly by year (*F*_(5,972)_ = 4.92, *p* < 0.001; *η*_p_^2^ = 0.02). Post hoc comparisons with the family-wise error rate controlled using the Tukey HSD method indicated that NBME scores were significantly lower in 2020 compared to 2018 (*t*(972) = 4.38, *p* < 0.001; *d* = 0.49), 2019 (*t*(972) = 4.17, *p* < 0.001; *d* = 0.47), and 2023 (*t*(972) = 3.26; *d* = 0.36,* p* < 0.05). All other comparisons were not significant (see Table 1). The estimated marginal means of NBME scores for all years are shown in Fig. 1.

**Table 1:** Post hoc comparisons of year difference for marginal means of NBME and H&P scores

Contrast	NBME		H & P	
	*t*-ratio	*p*-value	*t*-ratio	*p*-value
**2018 – 2019**	0.197	1.0000	− 1.582	0.6108
**2018 – 2020**	4.380	0.0002***	− 2.335	0.1811
**2018 – 2021**	1.638	0.5734	− 4.766	< 0.0001***
**2018 – 2022**	1.792	0.4711	− 3.961	0.0011**
**2018 – 2023**	1.165	0.8532	− 7.110	< 0.0001***
**2019 – 2020**	4.146	0.0005***	− 0.762	0.9738
**2019 – 2021**	1.426	0.7111	− 3.137	0.0217*
**2019 – 2023**	1.581	0.6113	− 2.355	0.1734
**2019 – 2023**	0.957	0.9313	− 5.450	< 0.0001***
**2020 – 2021**	− 2.765	0.0642	− 2.336	0.1805
**2020 – 2022**	− 2.573	0.1050	− 1.569	0.6190
**2020 – 2023**	− 3.256	0.0148*	− 4.622	0.0001***
**2021 – 2022**	0.169	1.0000	0.762	0.9738
**2021 – 2023**	− 0.482	0.9968	− 2.321	0.1867
**2022 – 2023**	− 0.648	0.9872	− 3.065	0.0271*

Asterisks indicate statistically significant *p*-values at the following levels: *p* < 0.05(*), *p* < 0.005(**), and *p* < 0.001(***). *p*-values were adjusted using the Tukey methods for comparing a family of six estimates

**Fig. 2:**
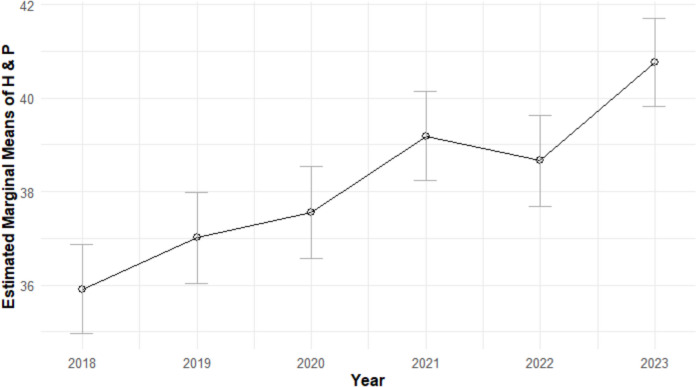
Estimated marginal means of H&P scores by year with 95% confidence intervals

Results of the second ANCOVA indicated H&P scores differed significantly by year, *F*_(5,966)_ = 12.35, *p* < 0.001; *η*_p_^2^ = 0.06, with post hoc comparisons shown in Table 1. Estimated marginal means of H&P scores for all years are shown in Fig. 2.

**Fig. 3:**
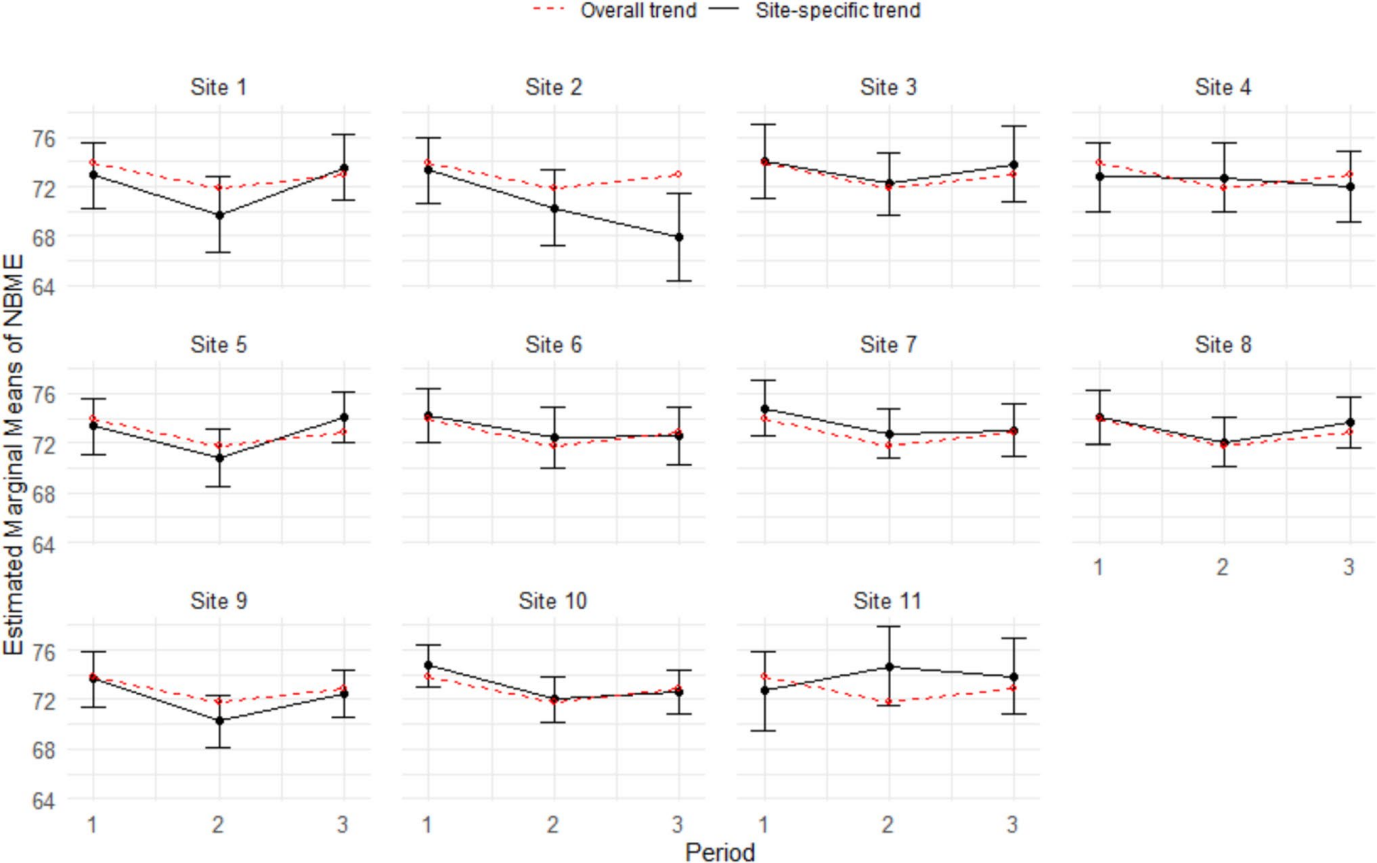
Estimated marginal means of NBME scores by period^☨^ with 95% confidence intervals for each site ^☨^Where period 1 is the regular clerkship schedule (2018 and 2019), period 2 is adapted clerkship schedule for COVID (2020 and 2021), and period 3 reflective of a return to regular clerkship schedule (2022 and 2023)

**Fig. 4:**
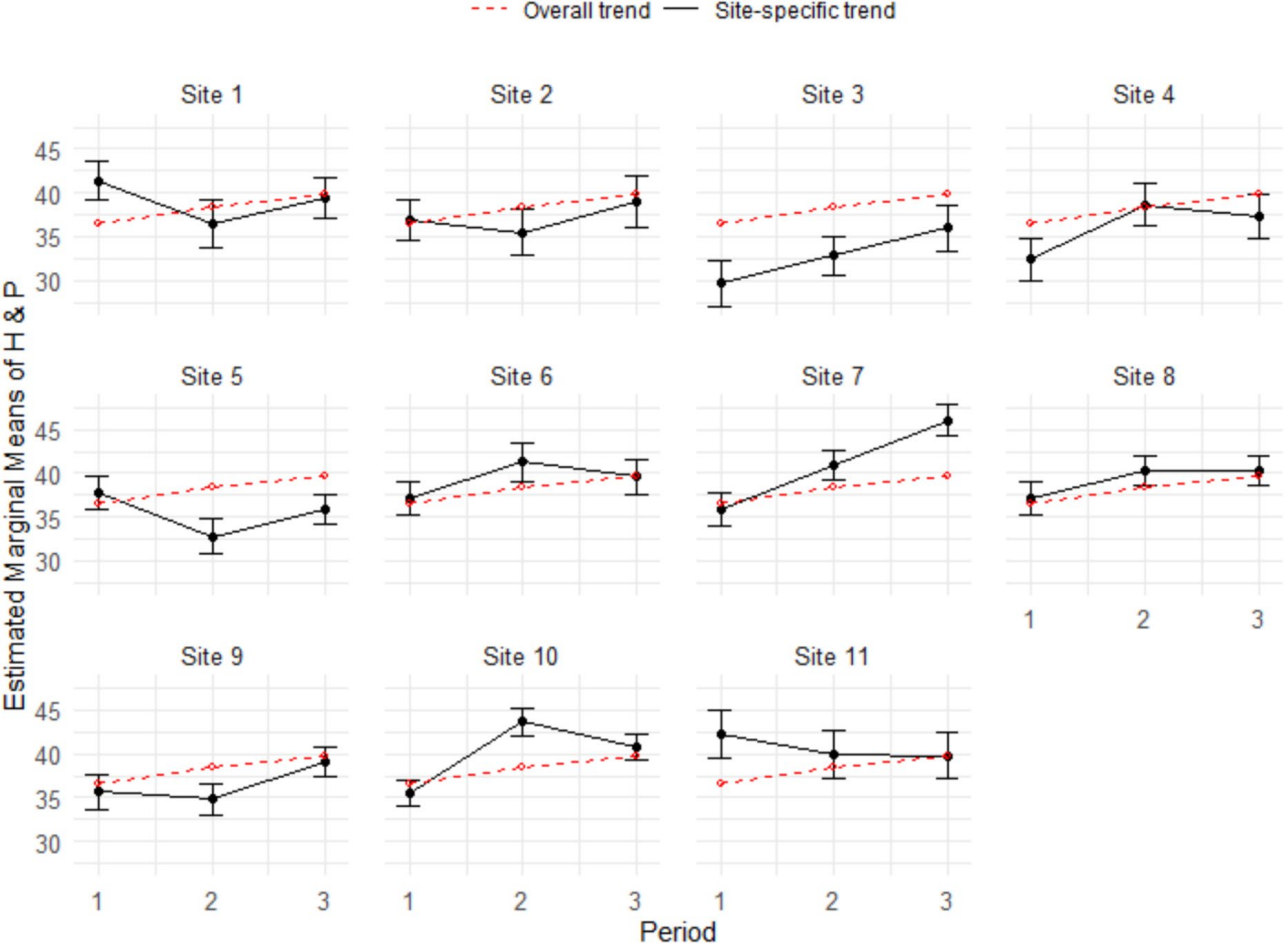
Estimated marginal means of H&P scores by period^☨^ with 95% confidence intervals for each site ^☨^Where period 1 is the regular clerkship schedule (2018 and 2019), period 2 is adapted clerkship schedule for COVID (2020 and 2021), and period 3 reflective of a return to regular clerkship schedule (2022 and 2023)

As can be seen by comparing Fig. 3 and Fig. 4, intersite consistency in NBME scores appeared to be greater than intersite consistency in H&P scores. As shown in Fig. 3, the confidence intervals for site-specific mean NBME scores included the overall means (represented by the dotted line) in all but one case (site 2, period 3). In contrast, Fig. 4 shows that nearly half of the confidence intervals for site-specific mean H&P scores did not include the overall means.

A Welch *t*-test of the standardized NBME and H&P scores revealed that the mean difference between site-specific and overall means was significantly greater for H&P scores (M_diff = 0.38) than for NBME scores (M_diff = 0.14),* t*(40) = 4.46, *p* < 0.001, indicating greater site-level variation in H&P scores (i.e., less intersite consistency) than NBME scores.

### The Relationship Between Round and Performance

For all students who completed their pediatric clerkship between 2018 and 2023, the regression analysis found a linear relationship between Round and NBME score that was significant (*B* = 0.72; *β* = 0.22, *p* < 0.001). Thus, for each increase in Round, NBME score increased by 0.72 points on average.

Results of a second regression analysis indicated that the linear relationship between Round and H&P scores was significant (*B* = 0.31; *β* = 0.12, *p* < 0.0001). For each increase in Round, H&P scores increased by 0.31 points.

A *z*-test compared the strength of the relationship between Round and NBME scores to the relationship between Round and H&P scores, while controlling for pre-clerkship NBME scores. The results indicated that the relationship between Round and NBME scores was significantly greater than the relationship between Round and H&P scores (*z* = 3.71, *p* < 0.001).

**Fig. 5:**
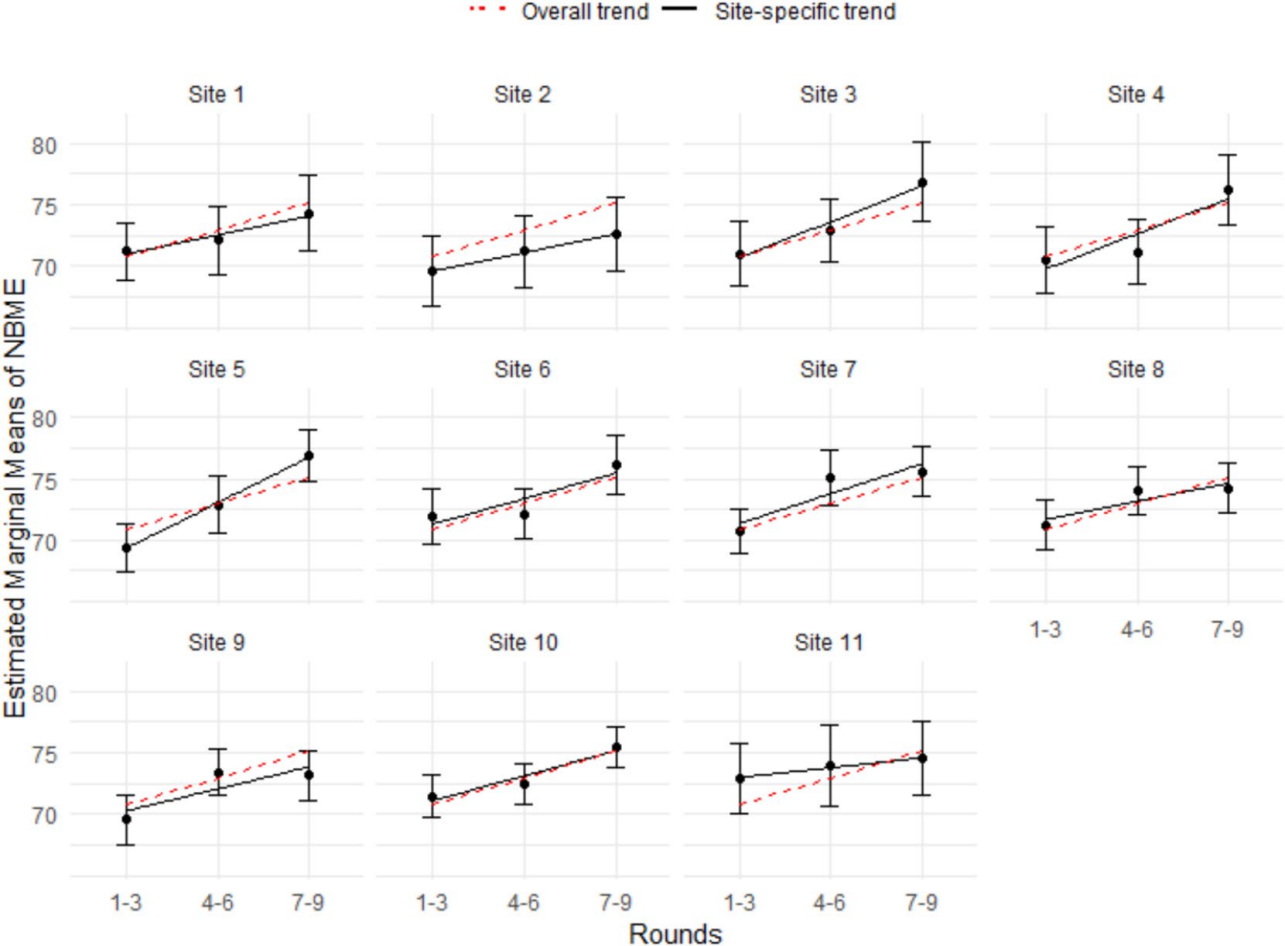
NBME means by grouped rounds with 95% confidence intervals for each site

**Fig. 6:**
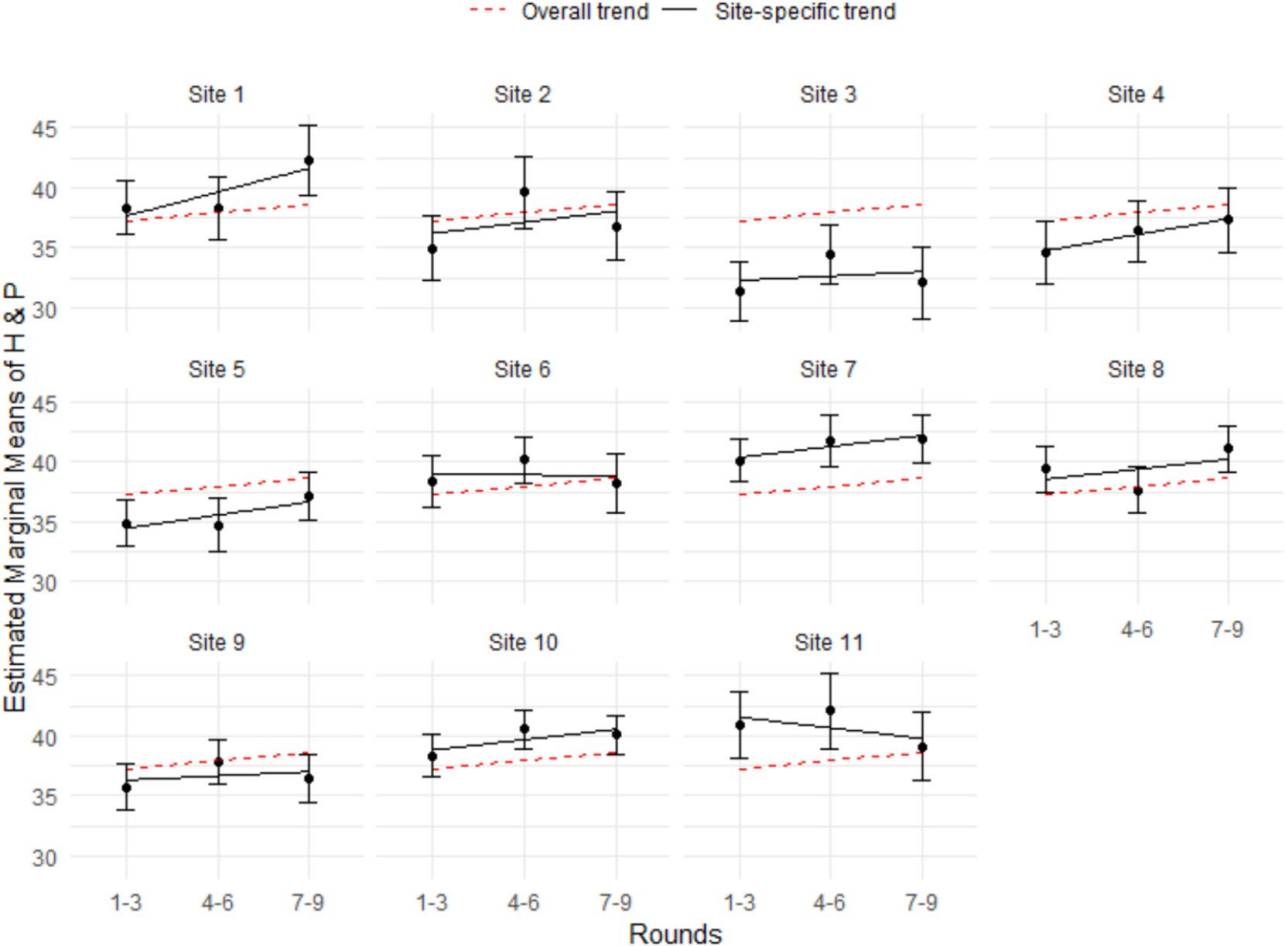
H&P means by grouped rounds with 95% confidence intervals for each site

The results of follow-up analyses investigating the relationship between Rounds and performance (see Figs. 5 and 6) revealed that site-specific NBME scores were more closely aligned with the overall NBME scores than site-specific H&P scores were with the overall H&P scores, indicating greater site-level variation in H&P scores compared to NBME scores. A Welch *t*-test conducted on the standardized NBME and H&P scores indicated that the mean difference between site-specific and overall means was significantly greater for H&P scores (M_diff = 0.35) than for NBME scores (M_diff = 0.12), *t*(40) = 5.46, *p* < 0.001.

The final set of analyses on potential interactive effects between year and Round on performance found no evidence of interactive effects for either the NBME or H&P outcomes. That is, from 2018 to 2023, there was no evidence that the relationship between the Round in which students completed the pediatric clerkship and their performance (i.e., NBME and H&P scores) was dependent upon the year.

## Discussion and Conclusion

The purpose of this study was to investigate potential impacts of the COVID-19 pandemic on medical students’ performance during their pediatric clerkship. Sub-analyses sought to determine if performance varied by pediatric clerkship site as well as by Round. Findings suggest curriculum modifications implemented to the pediatric clerkship during the COVID-19 pandemic (2020) may have adversely affected student performance on the NBME, although H&P scores have continued to increase. Sub-analysis revealed a positive correlation between Round and overall performance outcomes. However, inconsistencies in H&P scores across different clerkship sites suggest a need for enhanced standardization of the examination and/or its scoring criteria.

Medical students’ pediatrics NBME scores were significantly lower in 2020 compared to 2018, 2019, and 2023. This is consistent with the results from [9] that found a significant difference in medical students’ academic achievement during the pandemic compared to achievement from pre-pandemic. Our findings suggest that changes made to the USU pediatric clerkship curriculum during the COVID-19 pandemic (2020) may have impacted students’ performance on the NBME. In addition to an overall decrease in pediatric patient census during the pandemic [16, 17], contributing factors unique to USU medical students likely include a shortened clerkship and increased distance learning. Nonetheless, there are a number of studies that found no significant differences in medical students’ performance on the NBME during the pandemic [12, 18, 19]. Such variance in research outcomes signifies that there are additional factors that contributed to students’ performance during the COVID-19 pandemic. A number of studies have found that medical students experienced a variety of stressors during the pandemic that correlated to lower academic achievement [20, 21]. And, recent research out of Ecuador found that students were more likely to report their medical school curriculum was not fulfilling their learning objectives during the pandemic [22]. It is important to consider the broader impact of the pandemic on students’ well-being and learning environment. Researchers in this study suspect social isolation, disruptions to routines, and increased stress levels may have negatively affected cognitive function, reduced opportunities for peer collaboration, and diminished sense of community. These factors could have also contributed to the observed decline in NBME scores.

While students’ NBME scores decreased during the pandemic, H&P scores have consistently been rising since 2018 (see Fig. 2). These results may be due, in part, to a new grading policy for the H&P adopted by the pediatric clerkship in 2018, which permits students to resubmit the H&P to improve their score. Moreover, with reports of flexible grading, reduction of standardized assessment, and grade inflation during the pandemic [23], site directors may have been inclined to be less stringent in their grading.

Sub-analysis showed that alignment in NBME scores across the pediatric clerkship sites is consistent with prior research [14]. These results are encouraging and suggest that each pediatric clerkship site connected to the USU has been able to consistently, since 2015, ensure students are having comparable clinical experiences to prepare them for the NBME. On the other hand, there was noticeable variability in H&P scores (see Fig. 4) across pediatric clerkship sites. In addition to the revised grading practices mentioned above, there may be other factors that have contributed to the variability in H&P scores. Rubrics are lauded as valuable assessment tools that help students understand and fulfill expectations while improving validity and reliability in grading practices [24]. The P-HAPEE rubric, in particular, was shown to have an inter-rater reliability of 0.85 with 23 graders [25]. And while site directors are provided with annual training on the use of the P-HAPEE rubric, there may still be some inherent subjectivity in the site director’s explanation of the assignment to students as well as their interpretation of the rubric. These inconsistencies may be exacerbated by the transience of military personnel, resulting in higher turnover rates for site director positions at each MTF.

The sub-analysis investigating students’ NBME performance in relation to Round is consistent with findings from Fits et al. [26]. The more experience students have throughout the clerkship year, the better their overall performance. These findings are also consistent with the national trend for NBME scores, where the threshold for scoring within each percentile increases each quarter. Likewise, the USU Department of Pediatrics uses Honors-Pass-Fail grading, in which the criteria for earning Honors increase throughout the year. In Rounds 5–9, students must earn a higher score on the NBME than in Rounds 3–4 and Rounds 1–2. The positive correlation between Round and performance outcomes underscores the importance of clinical experiences for medical student development while reinforcing the decision to increase the criteria for obtaining Honors in the pediatric clerkship. The sub-analysis by site further reinforces the conclusions about inconsistency in H&P scores.

This study has some limitations. Due to a number of changes to assignments and grading policies within the Department of Pediatrics from 2018 to 2023, only two performance outcomes (i.e., NBME and H&P scores) remained as valid and comparable for our analysis. These changes further limited our ability to analyze students’ overall pediatrics clinical grade and capture a more complete spectrum of student performance during the pandemic. As previously mentioned, since 2018, the H&P assignment has allowed students to resubmit for a potential higher score; this may have inflated the means for the H&P. Lastly, given the Military Health System and the Uniformed Services University are nestled in a unique environment, results may not be generalizable to civilian institutions.

Overall, this study has revealed that while the COVID-19 pandemic may have impacted pediatric clerkship students’ NBME performance, their performance did not significantly vary by site, nor did it disrupt the positive correlation between Round and NBME performance. While this study focused specifically on the Pediatrics clerkship, it is plausible that similar pandemic-related impacts on student performance could be observed in other clerkships such as internal medicine, surgery, and Ob/Gyn. These specialties also experienced significant disruptions to clinical experiences and educational modalities during the pandemic, potentially affecting student learning and assessment outcomes. As civilian medical schools are forced to adopt multi-site clinical rotations at geographically diverse and distant clerkship sites, the USU may serve as a model for ensuring intersite consistency in NBME performance. Nevertheless, the collective findings of all analyses indicate the presence of deficiencies within the H&P and/or grading procedures and policies that warrant departmental intervention. The Department of Pediatrics at the USU is already taking steps to mitigate these variabilities, investigating the use of advanced technologies (e.g., artificial intelligence), and increased site director training, as well as improved guidance for students on assignment success criteria.

Future research should consider analyzing the trends in student performance before, during, and after the pandemic across all clerkships. Additionally, the USU researchers should consider a more holistic approach that analyzes potential student stressors and the effectiveness of the pediatric clerkship curriculum, as well as other military specific factors that may have impacted students’ performance, such as patient census at specific MTFs. Future research can further analyze site-specific elements (e.g., preceptors and patient complexity) that delve deeper into factors contributing to H&P score variability.

## Supplementary Information

Below is the link to the electronic supplementary material.

Supplementary file1 (DOCX 32.4 KB)

## Data Availability

The raw data cannot be shared, but aggregated results are available upon request.
