# Supplementary material for: Examining the Relationship Between Pediatric Clerkship Timing and Performance: COVID-19 Pandemic and Beyond
Source: Med Sci Educ. 2025 Mar 4;35(3):1503–11. doi: 10.1007/s40670-025-02339-2 (PMC12228625; doi:10.1007/s40670-025-02339-2)
Supplement: Supplementary file 1 — Supplementary file1 (DOCX 32.4 KB) [file 40670_2025_2339_MOESM1_ESM.docx]

**Supplementary Information**

**Appendix A: Pediatric History and Physical Exam Evaluation (P-HAPEE) rubric for grading the H&P exam**

**Pediatric Written History And Physical Exam Evaluation (P-HAPEE) Tool**

**Rater Instructions Brief**

- **Thank you for participating in H&P Scoring!** This document is a brief rubric introduction and/or refresher. We recommend you review additional materials (sample H&Ps with detailed answer key, detailed rater training booklet) at least once.

- H&P feedback is a **formative learning exercise** with shared goal of trainee clinical reasoning and documentation improvement. Fast turn-around time (48hrs or less) allows student to receive this feedback prior to obtaining and documenting additional H&P’s.

- **We recommend you start out by reading the assessment.** This puts the rest of the H&P into perspective and allows for a clearer idea as to whether the writer’s history and physical exam are hypothesis (assessment) driven.
- The rubric uses a 5-point scale with anchors to facilitate scoring and provide trainees with specific ideas for documentation improvement. Examples listed are meant to be illustrative not exclusive

- The rubric is criterion referenced. A “5” is a theoretical ideal H&P written for this particular patient. An experienced attending with time on his/her hands would write a “5” H&P.
  - A “3” describes someone who obtains a complete H&P, but who is gathering information first and formulating an assessment and differential later. A medical student is expected to achieve a score of 3 by the end of his/her third year.
  - A “5” describes someone whose H&P is both hypothesis (assessment) driven AND patient specific. A “5” clearly has a differential diagnosis in mind and who uses the H&P for hypothesis testing. The information obtained with each patient is highly individualized. A strong and motivated student who goes back to ask additional questions and/or perform additional physical exam maneuvers after obtaining the initial H&P might achieve a a “4” or “5” in some categories.
- Use an “intermediate” score of 2 or 4 for students who are approaching the higher category but not quite there
- In order to achieve a higher score, ALL the characteristics of the lower scores need to be met. To get a “5” on a given question a student needs to meet both the “3” AND the “5” criteria. For instance, a student who does an excellent job taking parent/family preferences and cost-effectiveness into account in his plan, but does NOT discuss discharge planning would get a maximum score of a “2” for the plan. Use the comment box and/or “things done well” to note the higher level skills.
- **Additional history (PMH, FH, SH, ROS).** Take ALL items into account when assigning a score. For instance a student who does NOT document a family history or whose ROS is inaccurate would receive a maximum score of “2” regardless of how well documented other items in this category are.
- **Problem Identification:** H&P formatting differs by both student and institution. Some students include a clearly enumerated problem list while others include identified problems within the assessment discussion. Both formats are acceptable. For patients with only a single hospital problem, 3 is a maximum score achievable for this item.
- **Assessment:** “big picture” synthesis of collected information leading to the formulation of a prioritized differential and identification of the most likely diagnosis**.** In formulating an assessment, author

**1. Selects** critical defining history AND physical exam/diagnostic study findings

2. **Synthesizes** these findings into medical concepts which typically involves

- translating lay language into medical terms. Eg: started today→acute; RR 60→tachypnea; pH 7.2→acidosis
- joining individual findings into syndromes. Eg: tachypnea, retractions, pH 7.2→respiratory distress
- characterizing findings using semantic qualifiers (descriptors that exist in divergent pairs). Eg: no relevant PMH/PHM significant for; acute/chronic; mild/severe; localized/diffuse; right sided/left sided, bilious/nonbilious, anemic/polycythemic, acidotic/alkalotic, hyperkalemic/hypokalemic, etc.
  - A “5” assessment begins with a summary statement (problem representation) that condenses this critical information into a 1-3 sentence summary.

Instead of a concise summary statement, some students “spread” critical findings throughout their assessment and differential diagnosis discussion. If they identify and synthesize critical information, even if it is not as concise as a strong problem representation, you can consider a score of 4.

- - Students often focus on history without taking physical exam and diagnostic study results into account, essentially restating the patient introduction. Inclusion of some defining physical exam and/or diagnostic study results is required for a score of “3.” A student who does NOT take physical exam/diagnostic study results into account in formulating an assessment could achieve a maximum score of 2 IF she or he appropriate identifies and “translates” history data. At times it is hard to tell whether the student is mentioning history or physical exam findings (eg. Wheezing). If the student documents the finding on physical exam, we’d give him credit for it.
  - **Assessment examples**
    - **A “1” assessment:** 6w old child of a 24yr old GBS, HIV, GC/chlamydia negative, RI mom with history of asthma. Pt with PMH significant for an extra digit being removed after birth who presents with throwing up that started around Memorial Day and with change from 8 to 4 wet diapers a day in the past week. Most likely diagnosis is….” Student restates the history without synthesis, includes irrelevant information has not “translated” information into medical terms, and does not include critical PE and/or diagnostic study information.
    - **A “3:” assessment.** “6w old former full term boy with past medical history significant for an extra digit being removed after birth who presents with 1.5 months of projectile NBNB emesis and is admitted due to dehydration with elevated Na and bicarb. Most likely diagnosis is…. Patient requires hospitalization due to…” Student still includes some irrelevant data (extra digit) but translates some critical hx information into medical terms (projectile, NBNB, dehydration), and incorporates some critical physical exam and diagnostic study results (elevated sodium and bicarb)
    - **A “5” assessment.** “6w old former full term male with progressive projectile nonbloody, nonbilious post-prandial emesis, with continued hunger and weight loss. Pt is moderately dehydrated with an olive-shaped right upper quadrant abdominal mass and laboratory findings significant for hypochloremic, hypokalemic metabolic alkalosis and prerenal kidney injury. Most likely diagnosis is…. Patient requires hospitalization due to ….
- **Plan:** Evaluate how the student addresses problem/s **he/she identified**. A student could potentially receive a “5” in this category even if his/her problem list is inaccurate or incomplete. Inclusion of decision making rationale (WHY a given antibiotic is chosen, WHY a study is ordered, what is the specific question for the consultant) as well as discharge/follow up planning is a requirement for a “3.”
- Please let us know if you have questions, suggestions, or comments at any time!

Author Name:

A medical student is expected to perform at a score of 3 or better by the end of his/her third year.

**History**

1. **Patient Introduction:** begins with the chief complaint (usually in patient’s/caregiver’s words), patient identifier, presence/absence of conditions directly relevant to the assessment, and reason for presentation

| 1 | 2 | 3 | 4 | 5 |
| --- | --- | --- | --- | --- |
| 1. Inaccurate , incomplete, and/or includes excessive irrelevant data |  | Accurate with most of the pertinent information included and most of the irrelevant data omitted |  | Concise and comprehensive |
| Notes: | | | | |

1. **History of Present Illness:** begins with the first change in health status related to the chief complaint and concludes at the time writer assumed patient care including reason for admission; identifies history source

| 1 | 2 | 3 | 4 | 5 |
| --- | --- | --- | --- | --- |
| 1. Inaccurate , incomplete, and/or includes excessive irrelevant data |  | Accurate, mostly organized sequence of relevant events with well-characterized symptoms (quality, severity, etc.) and most of the irrelevant data omitted |  | Hypothesis (assessment) driven, concise, comprehensive, organized. Includes PMH, FH, SH elements directly relevant to the differential and collateral history if indicated. |
| Notes: | | | | |

1. **Additional History: Past Medical History, Family History, Social History, Review of Systems**

| 1 | 2 | 3 | 4 | 5 |
| --- | --- | --- | --- | --- |
| 1. Inaccurate, incomplete, and/or poorly described |  | Accurate, complete, age-appropriate PMH, FH, SH, and ROS |  | Patient specific. Eg: developmental/nutritional screening; HEADSS for adolescents; seasonal influenza vaccine; close contact Tdap for infants, etc. |
| Notes: | | | | |

**Physical Exam and Diagnostic Studies**

1. **Vital Signs and Growth Parameters**

| 1 | 2 | 3 | 4 | 5 |
| --- | --- | --- | --- | --- |
| 1. Inaccurate and/or incomplete |  | Accurate with complete vital signs and some  age-appropriate growth parameters/percentiles (minimum wt and percentile) |  | All age-appropriate growth parameters/percentiles. Patient specific. Eg: preemie/syndrome specific growth chart; wt for length %; prior growth pattern; orthostatics; pain scale, etc. when appropriate |
| Notes: | | | | |

1. **Physical Exam**

| 1 | 2 | 3 | 4 | 5 |
| --- | --- | --- | --- | --- |
| 1. Inaccurate and/or incomplete |  | 1. Accurate, complete, age-appropriate physical exam with some expanded focus based on presenting symptoms. |  | 1. Hypothesis (assessment) driven. When appropriate, includes subtle positive and negative findings, comparison with past exams, and/or additional maneuvers that distinguish among diagnoses under consideration. Eg: dentition with suspected bulimia; acanthosis nigricans; comparison with prior liver size; psoas sign, etc. |
| Notes: | | | | |

1. **Diagnostic Studies**

 No diagnostic studies (current, past, or pending) relevant to presentation. Proceed to question 7.

| 1 | 2 | 3 | 4 | 5 |
| --- | --- | --- | --- | --- |
| Inaccurate, incomplete, and/or transcribed without appropriate attribution |  | Accurately reports pertinent positive and negative studies while omitting most of the irrelevant data |  | Accurately interprets pertinent positive and negative studies. When appropriate, includes review of prior studies, age/gender norms, calculations, and/or own review of diagnostic imaging. Eg: maternal labs; prior HbA1C; prior ejection fraction; Hb mean for age/gender; anion gap; Na correction for glu, etc. |
| Notes: | | | | |

**Information Synthesis and Clinical Reasoning**

1. **Problem Identification:** enumerated separately OR included within the assessment. Problems linked appropriately at highest diagnostic level based on available information (Eg. problems of fever, tachypnea, leukocytosis, RLL crackles and RLL CXR infiltrate linked under problem of RLL pneumonia)

| 1 | 2 | 3 | 4 | 5 |
| --- | --- | --- | --- | --- |
| 1. Absent, inaccurate, splits/joins problems inappropriately, and/or confuses systems and problems |  | Accurately identifies primary hospital problem/s at highest diagnostic level based on available information |  | Accurately prioritizes all active problems identified through history, physical exam, and diagnostic studies including secondary problems that should be addressed during hospitalization. Eg: second hand smoke exposure; underimmunized status; developmental delay; obesity; anemia, etc. |
| Notes: | | | | |

1. **Assessment:** “big picture” synthesis of collected information leading to the formulation of a prioritized differential and identification of the most likely diagnosis. Author (1) **Identifies** critical defining history AND physical exam/diagnostic study findings. (2) **Synthesizes** findings into medical terms and concepts (“started today”→acute; RR 60, retractions, pH 7.2→respiratory distress). (3) **Characterizes** findings using semantic qualifiers (no relevant PMH/PHM significant for; acute/chronic; mild/severe; acidotic/alkalotic).

| 1 | 2 | 3 | 4 | 5 |
| --- | --- | --- | --- | --- |
| Absent, unsupported, misses many critical findings, includes excessive irrelevant data, fails to include physical exam/diagnostic study findings, and/or restates findings without synthesis |  | Identifies some defining history AND physical exam/diagnostic study findings while omitting most of the irrelevant data. Uses some medical terms and semantic qualifiers to synthesize an assessment. |  | Selects critical defining history AND physical exam/diagnostic study findings. Uses appropriate medical terms and semantic qualifiers to synthesize an accurate and concise summary statement. |
| Notes: | | | | |

1. **Differential Diagnosis**

 No differential diagnosis relevant to presentation. This should be a rare event (Eg. pt presenting for scheduled chemotherapy). Proceed to question 9.

| 1 | 2 | 3 | 4 | 5 |
| --- | --- | --- | --- | --- |
| Absent, unsupported, and/or poorly described |  | Includes a prioritized differential while committing to a working diagnosis. Supports clinical reasoning with relevant history, physical exam, and diagnostic study elements. |  | Presents an accurate and concise differential by comparing/contrasting discriminating features of diagnoses under consideration. Includes a differential for secondary problem/s and/or refers to literature when appropriate. |
| Notes: | | | | |

1. **Plan:** diagnostic, therapeutic, patient/caregiver education, discharge, and follow-up

| 1 | 2 | 3 | 4 | 5 |
| --- | --- | --- | --- | --- |
| 1. Poorly described, unsupported, and/or does not match the problem list or assessment |  | Addresses most aspects of the identified problems while describing decision making rationale. Includes patient/caregiver education and discharge/follow-up plans when appropriate. |  | Accurately, concisely, and thoroughly addresses all identified problems. Considers patient/caregiver preferences, literature/practice guidelines, cost effectiveness, and/or contingency plans when appropriate. |
| Notes: | | | | |

Two things the author did well:

1.

2.

Two things the author should continue to work on:

1.

2.

“Stretch” goal:

1.

Overall H&P quality:

 Below expectations  Meets expectations  Exceeds expectations

Reprinted from Academic Pediatrics, 17, King MA, Phillipi CA, Buchanan PM, Lewin LO. Developing Validity Evidence for the Written Pediatric History and Physical Exam Evaluation Rubric, 68-73, 2017, with permission from Elsevier.
